# Antimicrobial Activity of Heterotrophic Bacterial Communities from the Marine Sponge *Erylus discophorus* (Astrophorida, Geodiidae)

**DOI:** 10.1371/journal.pone.0078992

**Published:** 2013-11-13

**Authors:** Ana Patrícia Graça, Joana Bondoso, Helena Gaspar, Joana R. Xavier, Maria Cândida Monteiro, Mercedes de la Cruz, Daniel Oves-Costales, Francisca Vicente, Olga Maria Lage

**Affiliations:** 1 Department of Biology, Faculty of Sciences, University of Porto, Porto, Portugal; 2 Interdisciplinary Centre of Marine and Environmental Research (CIMAR/CIIMAR), Porto, Portugal; 3 Centro de Química e Bioquímica e Departamento de Química e Bioquímica, Faculdade de Ciências, Universidade de Lisboa Campo Grande, Lisboa, Portugal; 4 CIBIO, Centro de Investigação em Biodiversidade e Recursos Genéticos, InBIO Laboratório Associado, Pólo dos Açores – Departamento de Biologia da Universidade dos Açores, Ponta Delgada, Portugal; 5 CEAB, Centre d'Estudis Avançats de Blanes, (CSIC), Blanes (Girona), Spain; 6 Fundación MEDINA, Centro de Excelencia en Investigación de Medicamentos Innovadores en Andalucía, Parque Tecnológico de Ciencias de la Salud, Armilla, Granada, Spain; University of Iowa Carver College of Medicine, United States of America

## Abstract

Heterotrophic bacteria associated with two specimens of the marine sponge *Erylus discophorus* were screened for their capacity to produce bioactive compounds against a panel of human pathogens (*Staphylococcus aureus* wild type and methicillin-resistant *S. aureus* (MRSA), *Bacillus subtilis*, *Pseudomonas aeruginosa*, *Acinetobacter baumanii*, *Candida albicans* and *Aspergillus fumigatus*), fish pathogen (*Aliivibrio fischeri*) and environmentally relevant bacteria (*Vibrio harveyi*). The sponges were collected in Berlengas Islands, Portugal. Of the 212 isolated heterotrophic bacteria belonging to *Alpha*- and *Gammaproteobacteria*, *Actinobacteria* and *Firmicutes*, 31% produced antimicrobial metabolites. Bioactivity was found against both Gram positive and Gram negative and clinically and environmentally relevant target microorganisms. Bioactivity was found mainly against *B. subtilis* and some bioactivity against *S. aureus* MRSA, *V. harveyi* and *A. fisheri*. No antifungal activity was detected. The three most bioactive genera were *Pseudovibrio* (47.0%), *Vibrio* (22.7%) and *Bacillus* (7.6%). Other less bioactive genera were *Labrenzia, Acinetobacter*, *Microbulbifer*, *Pseudomonas, Gordonia*, *Microbacterium*, *Micrococcus* and *Mycobacterium, Paenibacillus* and *Staphylococcus*. The search of polyketide I synthases (PKS-I) and nonribosomal peptide synthetases (NRPSs) genes in 59 of the bioactive bacteria suggested the presence of PKS-I in 12 strains, NRPS in 3 strains and both genes in 3 strains. Our results show the potential of the bacterial community associated with *Erylus discophorus* sponges as producers of bioactive compounds.

## Introduction

Due to their physico-chemical properties, which rarely exceed the biological tolerance limits, the oceans provide a safe environment for most living organisms [1]. However, the multiple beings that live in the marine environment had to develop survival strategies against other organisms with whom they have to compete for space and food. Sharing a common environment over a long evolutionary period also allowed the establishment of well-balanced associations between many of these organisms. A good example of these associations are the sponges that host a significant microbiome which can reach up to 40–60% of the total sponge biomass and densities of 10^8^ to 10^10^ bacteria per gram of sponge wet weight. These values can exceed seawater concentrations by two to three orders of magnitude [2]–[5].

Natural bioactive compounds have been used since the beginning of traditional medicine [6]. They are present in all kinds of life forms and are produced by the secondary metabolism of organisms. Secondary metabolites include terpenoids, alkaloids, polyketides, peptides, shikimic acid derivates, sugars, steroids and a large mixture of biogenesis metabolites [7]. Sponge symbionts are fundamental in host defence against predators due to the production of biologically active secondary metabolites. These natural products can show antibacterial, antifungal, antiviral, antiprotozoal, anthelmintic, anti-inflammatory, antitumor, immunosuppressive, neurosuppressive properties and can also possess activities for the treatment of cardiac, respiratory and gastrointestinal diseases [8]. Sponges are the “gold mine” organisms for natural product isolation (over 30% of the products) in the marine environment [9]. The search for new drugs, especially antibiotics, is important due to the increase of bacterial resistance to existing antibiotics.

The true origin of many of these bioactive molecules is uncertain. The production of secondary metabolites could be due to the cooperation between sponges and symbionts, only to the symbionts or to the sponges [10]; [11]. A microbial origin got support from the occurrence of structurally similar substances in unrelated sponges (see Laport *et al*. [8]). The fact that these substances may be produced by microbes could allow their sustainable and unlimited production *in vitro*. This is hardly the case with sponges. As the sponge bioactivity may in fact be due to their microbiome, these organisms became the subject of many works.

Secondary metabolites possess complex structures and involve unusual biochemistry. Two families of enzymes, the polyketide synthases (PKS) and the nonribosomal peptide synthetases (NRPS), are of particular importance in the production of various secondary metabolites many of which are important drugs [12]. Both PKSs and NRPSs can be conceptualized as enzymatic “assembly lines” composed of functional modules [13].

The discovery of new biosynthetic pathways encoding genes of secondary metabolites opens the possibility of heterologous production and the genetic manipulation of the gene cluster to obtain new natural products [14]. Metagenomic analysis of the prevalence and presence of PKS and NRPS genes are being studied to improve the search of new bioactive compounds in sponges (*e.g.* Schirmer *et al*. [15]).

Sponges belonging to the genus *Erylus* (Astrophorida, Geodiidae), are well known as producers of saponins and other oligoglycosides [16]–[27]. These natural products have been reported to exhibit a wide spectrum of biological activities which include selective thrombin receptor antagonist activity and functional activity in a platelet aggregation assay [19], immunopressive activity [28], inhibition of neuraminidase from the bacterium *Clostridium perfringens* [21] and antitumor and antifungal properties [16]; [23]; [24]. Alcoholic extracts of *Erylus deficiens* Topsent, 1927 from the Mediterranean sea showed antibacterial activity against the marine bacteria *Alteromonas luteo-violacea* and 8 terrestrial bacteria (*Staphylococcus epidermidis*, *Sarcina lutea*, *Bacillus subtilis*, *Micrococcus luteus*, *Serratia marcescens*, *Enterobacter sp*., *Proteus morganii* and *Proteus mirabilis*) [29]. Antimicrobial activity against *Bacillus subtilis*, *Escherichia coli* and *Candida albicans* was observed in the extracts of *Erylus lendenfeldi* Sollas, 1888 collected in the Red Sea [22]. In addition, multiple ecological functions have also been attributed to these molecules. Furthermore, it was shown that the sponge *Erylus formosus* Sollas, 1886 contained sufficiently high concentrations of erylosides to protect itself against predatory fishes, bacterial settlement, and fouling [30]–[31]. These natural compounds have been isolated from different species of *Erylus* as well as from different geographic locations [23]–[27]. Another species, *E. discophorus* Schmidt, 1862, was reported to have haloperoxidase with iodo- and bromoperoxidases activities [32].

In this study we aimed to assess the bioactive potential of bacteria isolated from the marine sponge *E. discophorus* collected in Berlengas Islands against a panel of pathogenic and environmental microorganisms. Antimicrobial bioactivity was detected in 31% of the 212 isolated heterotrophic bacteria from two specimens of *E. discophorus* and the presence of PKS-I and NRPSs genes was detected in several isolates showing the biotechnological potential of these bacteria.

## Materials and Methods

### 1. Biological material

The 212 bacteria (here designated test bacteria) under study were isolated from two specimens of the sponge *E. discophorus* (named Berg01 and Berg02) that were sampled nearby, at 15 m by scuba diving in the Berlengas Islands a protected area of UNESCO located off the W coast of Portugal (N39° 28′ 47″; W9° 32′ 78″). The authors thank Reserva Natural das Berlengas (Instituto da Conservação da Natureza e da Biodiversidade –ICNB) for the sponges samples harvesting permission. Voucher samples of the sponges were preserved in 90% ethanol for taxonomic identification and deposited in the Biology Department's zoological collection of the University of the Azores (DBUA.Por). Specimens were identified from the analysis of general external and internal morphological characters, i.e. shape, type, size and arrangement of skeletal structures (spicules) following the Systema Porifera classification system [33].

The bacterial isolation was performed inoculating serial dilutions (10^−1^ to 10^−6^) of the homogenized from the sponges in heterotrophic media. After isolation in pure culture, bacteria were cultivated in Marine Broth (Becton Dickinson, MB) in the dark at 26°C. Their taxonomic identification was based on the analysis of the 16S rRNA gene by direct colony PCR or with template DNA extracted by the boiling method. A loop full of bacterial culture was resuspended in 200 µl of distilled deionized H_2_O and subjected for 10 min to 100°C, cooled on ice and the extracted DNA amplified with the universal primers 27f and 1492r [34] in 50 µl of PCR mixture (1× PCR buffer; 1.5 mM MgCl2; 1 unit of GoTaq Flexi DNA Polymerase; 200 µM of each deoxynucleoside triphosphate (dNTPs); 2 µM of each primer). The PCR program was performed in a MyCycler™ Thermo Cycler (Bio-Rad) and consisted in an initial denaturing step of 5 min at 95°C; 30 cycles of 1 min at 94°C; 1 min at 52°C; and 90 s at 72°C; and a final extension of 5 min at 72°C. PCR products were visualized after electrophoresis in a 1.2% agarose gel in 1X TBE buffer. The PCRs amplicons were sequenced at MACROGEN (Korea). Sequences were assembled with Vector NTI 10.1 and classified in the Ribosomal Database Project [35].

The isolated *Erylus* bacteria were screened for their capacity to produce bioactive compounds against a panel of varied target microorganisms. This includes human pathogens, a fish pathogen and an environmentally relevant bacterium. In the Janus assays, *Bacillus subtilis* (ATCC6633), *Staphylococcus aureus* MRSA, *Acinetobacter baumannii* and *Candida albicans* were tested while in the Duetz assays *Pseudomonas aeruginosa* PAO1, *Acinetobacter baumannii*, *Vibrio harveyi* (CECT 525), *Aliivibrio fischeri* (CECT 524), *Bacillus subtilis* (ATCC6633), *Staphylococcus aureus* wild type Smith strain, *Staphylococcus aureus* MRSA, *Candida albicans*, *Aspergillus fumigatus* (ATCC46645) and (Δ*αkuB* ^KU80^) were used.

### 2. Screening assays for antibacterial and antifungal activity

The search for bioactivity was performed with the 212 bacteria isolated from *E. discophorus*. The isolates were initially fermented in 96-deep well Duetz plates [36] with 800 µl of MB media in each well for 3 days at 28°C and 220 r.p.m. After incubation, 400 µl of each culture were transferred to new 96-deep wells plates containing 400 µl of a solution of Sea Salts (4%) and Glycerol (40%) and kept at −28°C. These plates were designated as the Master Plates (MP). The MPs were then used in a replication procedure previously described [36] to generate fresh inocula (MB medium, 2 days, 28°C, 220 rpm, 70% humidity). The inocula thus generated were employed to seed all subsequent micro-fermentations carried out in both *Janus* and Duetz plates.

#### 2.1 *Janus* plates assays

This double-faced plated assay optimized by Fundación MEDINA [37] is based in exclusively designed plates by Nunc with two sides, which allow culture growth on the opposite layers in solid media. In order to maximize the number of potential active secondary metabolites produced, 5 different media were chosen to carry out the miniaturized fermentations: Marine Broth (2216 Difco), Medium F (0.015 g K_2_HPO4; 0.2 g CaCl_2_; 0.75 g KCL; 23.4 g NaCl; 7 g MgSO4; 1 g Mannitol; 1 g Yeast extract; 1 g Peptone; 16 g Agar; 1 ml Hutner's basal salts [38] and 999 ml ddH_2_O), R2A (218263 - Difco) and saline (supplemented with 3% of SeaSalts of Sigma) R2A and Starch Agar (Difco).

On the top layer of the *Janus* plates the test bacteria were inoculated employing a replication procedure previously described [36] and incubated for 3 days at 28°C and 70% of humidity. Subsequently, the inoculated media with the target microorganisms were added on the opposite side thus forming the assay layer. The double-faced *Janus* plates were incubated at 37°C for 20–24 h. A search of inhibition zones indicative of antibacterial or antifungal activity was then carried out [37]. This *in vitro* screening assay was performed against a panel of Gram positive bacteria (*Bacillus subtilis* (ATCC6633) and *Staphylococcus aureus* MRSA), Gram negative bacteria (*Acinetobacter baumannii*) and the yeast *Candida albicans*. All target organisms were pre-inoculated and grown at 28°C, 220 r.p.m. for 12 h and 70% of humidity. Pre-inoculum and inoculum medium used to grow *A. baumannii* and *B. subtilis* was Luria Broth (LB) (Miller's, Invitrogen). For *C. albicans* the pre-inoculum medium was Sabouraud Dextrose Broth (SDB) and the inoculum medium was YNBD [37]. The pre-inoculum and inoculum medium prepared for the overnight growth of *S. aureus* MRSA was Brain Heart Infusion (BHI). Inocula absorbance for all target microorganisms was adjusted to a final 600 nm optical density (OD) of 0.4 before being placed in the *Janus* plates. Non-inoculated medium was used as negative control.

Tests of growth interference of the target microorganisms with saline media were performed in advance to rule out possible interferences.

#### 2.2 Microfermentations in 96-deep well plates

MPs were used to carry out microfermentations in 96-deep well plates (here designated by Duetz system assay) following the approach described by Duetz *et al*. [36]. For this assay, besides the 5 media already specified for the *Janus* plates, half saline concentration of the media were also tested. The inoculated Duetz plates were incubated (1 mL) for 5 days at 28°C, 220 r.p.m. and 70% of humidity. Bacterial broth were then subjected to an organic extraction with 800 µl acetone and 40 µl DMSO per well. The plates were incubated for 1 h at 220 r.p.m. and then transferred to a vacuum centrifuge (GeneVac HT-24) in order to reduce the final volume to 400 µl (2 Whole Broth Equivalent). The supernatants of the extracts solutions were transferred to 96-deep well plates. The organic extracts were then assayed against the Gram-negative *Pseudomonas aeruginosa* PAO1, *Acinetobacter baumannii*, *Vibrio harveyi* (CECT 525) and *Aliivibrio fischeri* (CECT 524); and the Gram-positive *Bacillus subtilis* (ATCC6633), *Staphylococcus aureus* wild type Smith strain and *Staphylococcus aureus* MRSA. Antifungal assays were also prepared against the yeast *Candida albicans* and *Aspergillus fumigatus* (ATCC46645) and (Δ*αkuB* ^KU80^). Unless specified negative controls were always uncultivated culture medium.

For the screening assays against *Acinetobacter baumannii*, *S. aureus* (Smith), *Pseudomas aeruginosa* PAO1 and *Candida albicans*, overnight cultures grown in liquid Luria Broth (Miller's media) at 37°C and 220 r.p.m. were measured at an absorbance of 612 nm for *A. baumannii* and *S. aureus* (Smith) and 600 nm for *P. aeruginosa*. The inocula in LB media were adjusted to an OD of 5×10^5^ for *A. baumannii* and to a cell concentration of 2.5×10^5^ CFU/ml for *S. aureus* (Smith) and 5×10^5^ CFU/ml for *P. aeruginosa*. A suspension of *C. albicans* in medium RPMI (40 ml 1 M HEPES; 36 ml 50% glucose; 6.7 g Yeast Nitrogen Broth without amino acids; RPMI a bottle; adjust pH to 7.1 and 0.22 µm sterilized) was adjusted to 0.250 at 660 nm. This suspension was then diluted 1/10.

For the liquid screens, Nunc plates (LTC-330) were filled as follows: 10 µl of each extract plus 90 µl of inoculum in 80 wells; 10 µl of a series of antibiotic concentrations in the range of the minimum inhibitory concentration plus 90 µl of inoculum (positive and negative controls) in 8 wells; 100 µl (90 µl of LB media +10 µl DMSO 20%) as blank control in 4 wells; and 100 µl of the inoculum for the assessment of the total microorganism growth. The antibiotics used against *A. baumannii* were, as positive controls, 0.3125, 0.625, 12.5 and 25 µg.mL^−1^ rifampicin and, as negative controls, 7.8125, 15.625, 31.25 and 62.5 µg.mL^−1^ amphotericin B. In the case of *P. aeruginosa* the positive controls were 1.9625, 3.925, 7.85 and 15.7 µg.mL^−1^ ciprofloxacin and the negative controls were 7.8125, 15.625, 31.25 and 62.5 µg.mL^−1^ amphotericin B. In the assays performed with *S. aureus* (Smith) the positive controls used were serially diluted between 0.5×10^−7^ to 5 mg.mL^−1^ penicillin G and with *C. albicans* the antibiotics used were as positive controls 7.8125, 15.625, 31.25 and 62.5 µg.mL^−1^ amphotericin B and as negative controls 0.039, 0.078, 0.156 and 0.312 mg.mL^−1^ penicillin G.

The plates were incubated at 37°C for 20 h under humid conditions. The absorbances were measured at 612 nm in a Tecan ULTRAEVOLUCION before and after incubation. To confirm the results, 30 µl of a 0.02% resazurin stock solution was added to each well (100 µl) and incubated for 2 h. Changes in colour from blue (growth inhibition corresponding to detection of bioactivity) to pink (no growth inhibition corresponding to no detection of bioactivity) and fluorescence readings radiated from the resazurin were measured in a Perkin Elmer VICTOR2 multi-function fluorometer. All ODs and fluorescence measurements were analysed using the Genedata Screener software.

For the screening assays against *Aspergillus fumigatus* ATCC46645 and Δ*αkuB* ^KU80^, cultures of both *A. fumigatus* were prepared in medium RPMI with 0.002% resazurin from a stock spore suspension in Tween saline buffer to a final concentration of 2.5×10^4^ conidia/ml as described by Monteiro *et al*. [39]. The assays were carried out as previously described, using as positive controls 0.5, 1.0, 2.0 and 4.0 µg.mL^−1^ amphotericin B. The plates were incubated for 30 h at 37°C and, then, the fluorescence was recorded in a Perkin Elmer VICTOR2^2^™ Multi-function fluorometer.

For the screening assays against *Staphylococcus aureus* MRSA, an overnight culture of *S. aureus* MRSA in 10 ml of liquid Brain Heart Infusion (BHI) medium was incubated at 37°C and 220 r.p.m. The OD of the culture measured at 660 nm was adjusted to 0.2 and used to inoculate BHI agar medium (3 ml of the adjusted inoculum per 100 ml of medium) which was then distributed (30 ml) in OmnyTray plates. Disposable 96 pin trays were used to generate 96 wells in each of the BHI agar plates in which, subsequently, 10 µl of each extract in each well were dispensed. Alternatively extracts were distributed in BHI agar plates without wells. Ten µl of 0.5 mg.mL^−1^ kanamycin was used as positive control. Plates were then incubated at 37°C for 20 h and zones of inhibition (ZOI) were measured. Any extract producing a visibly discernible ZOI, regardless of zone quality, was considered to be positive.

For the screening assays against *Bacillus subtilis* (ATCC 6633), a culture of *B. subtilis* was prepared using 1 ml of spore suspension/1 L medium (23 g/L of Nutrient Agar and 2 g/L of yeast extract) that had been previously sonicated for 3 min. The assay was performed in a similar way to the one used against *S. aureus* MRSA and the positive control was 150 µg/ml tunicamycin.

Screening assays against *Vibrio harveyi* CECT 525 and *Aliivibrio fischeri* CECT 524 were carried out in a cell density optimized agar assay. Ten ml of Luminous Medium [40] were inoculated with a loop of the pure *V. harveyi* and *A. fischeri*, incubated at 25°C, 220 r.p.m. for 12 h and the optical density adjusted to 0.3 for *V. harveyi* and 0.4 for *A. fischeri* at 600 nm and, subsequently, both diluted by 1/10. The assay was performed in a similar way to the one used against *S. aureus* MRSA and the positive control consisted on 0.256 mg/ml chloramphenicol. The cultures were incubated at 25°C for 24 h. Inhibition was detected with the presence of non-phosphorescent halos in a dark-room.

### 3. Search of PKS and NRPS genes

The molecular analysis of the genes PKS-I and NRPS involved in the production of secondary metabolites was investigated in the 59 of the bioactive bacteria. Genomic DNA was extracted with an E.Z.N.A. bacterial KIT from OMEGA. Specific degenerated primers MDPQQRf and HGTGTr [41] and DKf and MTr [42] were used for PCR amplification of PKS-I and NRPS genes, respectively. A total of 25 µl of PCR mixture (1× PCR buffer with 1.7 mM MgCl2; 0.8 unit of Go Taq DNA Polymerase; 0.2 mM of each dNTPs; 0.1 µM of each primer and 5 µl DNA template) was used. The PCR program for the genes PKS-I and NRPS was the same and consisted of an initial denaturing step of 5 min at 95°C; 11 cycles of 1 min at 95°C; 30 s at 60°C and 1 min at 72°C, with the annealing temperature reduced by 2°C per cycle, followed by 30 cycles of 95°C for 1 min, 40°C for 30 s and 72°C for 1 min with a final extension of 10 min at 72°C. The PCR programs were performed in an MyCycler™ Thermo Cycler (Bio-Rad) and the PCR products were visualized in VWR GenoPlex after electrophoresis in a 1.2% agarose gel in 1X TAE buffer.

## Results and Discussion

The oceans, an almost endless source of microbial diversity, are the habitat of organisms such as sponges that harbour a large microbial diversity with important biosynthetic potential due to their secondary metabolites profiles [43]. The analysis of sponge symbionts in pure cultures is an advantage for the performance of bioactive screening assays [44] and is the most direct method for the large-scale production of bioactive compounds [45]. The two specimens of *Erylus discophorus* collected in Berlengas (Berg01 and Berg02) allowed the isolation of a large number of heterotrophic bacteria (212 isolates) of which 31% (66 isolates) showed bioactivity. Of the screened bacteria for bioactivity and based on the 16S rDNA gene analysis, 57% (n = 120) were *Alphaproteobacteria*, 21% (n = 45) *Gammaproteobacteria*, 16% (n = 34) *Actinobacteria* and 6% (n = 13) *Firmicutes*.

Bioactivity was observed against one or more of the target microorganisms tested. The majority was active against *Bacillus subtilis* (87.9%) and at a lower percentage against *Staphylococcus aureus* MRSA (10.6%), *Aliivibrio fischeri* (9.1%) and *Vibrio harveyi* (6.1%). Bacteria with bioactivity against both *B. subtilis* and *S. aureus* MRSA represented 9.1% and against both *A. fischeri* and *V. harveyi* accounted for 4.6%. No bioactivity was observed against *P. aeroginosa*, *A. baumannii*, *S. aureus* wild type, *C. albicans* and *A. fumigatus*.

The taxonomic affiliation of all bioactive isolates is provided in Table 1.

**Table 1:** Taxonomic affiliation of the bioactive bacteria isolated from *E. discophorus*.

Isolate	Closest strain; Accession no.	Similarity	Genera	Phylum/Class
Berg02_22.2	*Gordonia sp*. DEOB200; AY927227	100,0%	*Gordonia*	Actinobacteria
Berg02_29	*Gordonia sp*. CNJ786 PL04; DQ448772	98.6%	*Gordonia*	Actinobacteria
Berg02_78	*Gordonia terrae*; 3269aBRRJ; FJ200386	99.6%	*Gordonia*	Actinobacteria
Berg02_79	*Microbacterium sp*. M63-2; EF061897	98.8%	*Microbacterium*	Actinobacteria
Berg02_79a	*Microbacterium sp*. M63-2; EF061897	99.7%	*Microbacterium*	Actinobacteria
Berg02_11	*Micrococcus sp*. LJY5; EU379020	99.7%	*Micrococcus*	Actinobacteria
Berg02_26	*Micrococcus luteus*; KCL-1; DQ538135	98.7%	*Micrococcus*	Actinobacteria
Berg01_46	*Mycobacterium sp*. JL838; DQ985057	96.9%	*Mycobacterium*	Actinobacteria
Berg02_114.2	*alpha proteobacterium* CRA 40S; AY562568	96.4%	*Labrenzia*	Alphaproteobacteria
Berg01_7	*Pseudovibrio ascidiaceicola*	100.0%	*Pseudovibrio*	Alphaproteobacteria
Berg01_9	*Pseudovibrio ascidiaceicola* (T); F423( = NBRC 100514); AB175663	99.8%	*Pseudovibrio*	Alphaproteobacteria
Berg01_16	*sponge bacterium* Isolate3; AY948383	98.3%	*Pseudovibrio*	Alphaproteobacteria
Berg01_33	*alpha proteobacterium* CRA 3GB; AY562562	96.2%	*Pseudovibrio*	Alphaproteobacteria
Berg01_50	*alpha proteobacterium* CRA 3GB; AY562562	99.6%	*Pseudovibrio*	Alphaproteobacteria
Berg01_68	*Pseudovibrio sp*. B411; FN295808	98.1%	*Pseudovibrio*	Alphaproteobacteria
Berg01_77	*Pseudovibrio sp*. PV4; EU768841	98.9%	*Pseudovibrio*	Alphaproteobacteria
Berg02_8.1	*alpha proteobacterium* CRA 3GB; AY562562	98.6%	*Pseudovibrio*	Alphaproteobacteria
Berg02_8.3	*sponge bacterium* Isolate1; AY948382	93.7%	*Pseudovibrio*	Alphaproteobacteria
Berg02_9.1	*alpha proteobacterium* CRA 3GB; AY562562	99.4%	*Pseudovibrio*	Alphaproteobacteria
Berg02_10.1	*Pseudovibrio sp*. PV4; EU768841	99.2%	*Pseudovibrio*	Alphaproteobacteria
Berg02_10c	*alpha proteobacterium* CRA 3GB; AY562562	99.3%	*Pseudovibrio*	Alphaproteobacteria
Berg02_13.1	*Pseudovibrio sp*. PV4; EU768841	99.2%	*Pseudovibrio*	Alphaproteobacteria
Berg02_17	*Pseudovibrio sp*. PV4; EU768841	98.7%	*Pseudovibrio*	Alphaproteobacteria
Berg02_18	*sponge bacterium* Isolate1; AY948382	96.0%	*Pseudovibrio*	Alphaproteobacteria
Berg02_36	*alpha proteobacterium* CRA 3GB; AY562562	97.1%	*Pseudovibrio*	Alphaproteobacteria
Berg02_39.1	*sponge bacterium* Isolate3; AY948383	98.3%	*Pseudovibrio*	Alphaproteobacteria
Berg02_39.3	*sponge bacterium* Isolate1; AY948382	99.5%	*Pseudovibrio*	Alphaproteobacteria
Berg02_40	*sponge bacterium* Isolate1; AY948382	98.4%	*Pseudovibrio*	Alphaproteobacteria
Berg02_41	*alpha proteobacterium* CRA 3GB; AY562562	98.8%	*Pseudovibrio*	Alphaproteobacteria
Berg02_50	*alpha proteobacterium* CRA 3GB; AY562562	96.4%	*Pseudovibrio*	Alphaproteobacteria
Berg02_51	*alpha proteobacterium* CRA 57G; AY562561	96.6%	*Pseudovibrio*	Alphaproteobacteria
Berg02_54.1	*Pseudovibrio sp*. PV4; EU768841	99.3%	*Pseudovibrio*	Alphaproteobacteria
Berg02_56.2	*alpha proteobacterium* CRA 3GB; AY562562	98.6%	*Pseudovibrio*	Alphaproteobacteria
Berg02_57	*sponge bacterium* Isolate3; AY948383	98.3%	*Pseudovibrio*	Alphaproteobacteria
Berg02_58	*Pseudovibrio sp*. PV4; EU768841	98.8%	*Pseudovibrio*	Alphaproteobacteria
Berg02_61	*Pseudovibrio ascidiaceicola* (T); F423( = NBRC 100514); AB175663	97.4%	*Pseudovibrio*	Alphaproteobacteria
Berg02_63	*sponge bacterium* Isolate1; AY948382	99.4%	*Pseudovibrio*	Alphaproteobacteria
Berg02_65	*Pseudovibrio ascidiaceicola* (T); F423( = NBRC 100514); AB175663	100,0%	*Pseudovibrio*	Alphaproteobacteria
Berg02_141	*sponge bacterium* Isolate1; AY948382	99.6%	*Pseudovibrio*	Alphaproteobacteria
Berg02_188	*sponge bacterium* Isolate3; AY948383	98.0%	*Pseudovibrio*	Alphaproteobacteria
Berg01_47	*Bacillus circulans*	99.0%	*Bacillus*	Firmicutes
Berg01_48	*Bacillus circulans* (T); AY043084	97.9%	*Bacillus*	Firmicutes
Berg01_114	*Bacillus sp. enrichment culture clone* SYW22; FJ601652	99.9%	*Bacillus*	Firmicutes
Berg02_107	*Bacillus megaterium*; MO31; AY553118	100.0%	*Bacillus*	Firmicutes
Berg02_161a	*Bacillus sp*. RS654(2010); GU968484	99.6%	*Bacillus*	Firmicutes
Berg01_119	*Paenibacillus sp*. 1 GUW; EU496552	99.6%	*Paenibacillus*	Firmicutes
Berg02_117	*Sporosarcina luteola*	99.9%	*Sporosarcina*	Firmicutes
Berg02_67	*uncultured bacterium*; nbu377a05c1; GQ035792	99.0%	*Staphylococcus*	Firmicutes
Berg02_21	*Acinetobacter sp*. PmeaMuc16; EU249988	98.0%	*Acinetobacter*	Gammaproteobacteria
Berg02_116	*Microbulbifer cystodytense*; C1; AJ620879	97.4%	*Microbulbifer*	Gammaproteobacteria
Berg02_77	*Pseudomonas sp*. CJ11064; AF500211	99.8%	*Pseudomonas*	Gammaproteobacteria
Berg02_5	*Vibrio sp*. Mel 35; AJ582806	100.0%	*Vibrio*	Gammaproteobacteria
Berg02_6	*Vibrio sp*. Mel 35; AJ582806	100.0%	*Vibrio*	Gammaproteobacteria
Berg02_16	*Vibrio sp*. Mel 35; AJ582806	100.0%	*Vibrio*	Gammaproteobacteria
Berg02_19	*Vibrio sp*. BBsea_03; JF792677	100.0%	*Vibrio*	Gammaproteobacteria
Berg02_22	*Vibrio alginolyticus*; UDC324; GQ245913	98.5%	*Vibrio*	Gammaproteobacteria
Berg02_23	*Vibrio splendidus*; DCM1; AY227706	99.9%	*Vibrio*	Gammaproteobacteria
Berg02_24	*Vibrio sp*. Mel 35; AJ582806	97.9%	*Vibrio*	Gammaproteobacteria
Berg02_28	*Vibrio alginolyticus*; UDC324; GQ245913	98.8%	*Vibrio*	Gammaproteobacteria
Berg02_45	*Vibrio splendidus*; 01/114; AJ874362	99.8%	*Vibrio*	Gammaproteobacteria
Berg02_47	*Vibrio sp*. BBsea_03; JF792677	99.9%	*Vibrio*	Gammaproteobacteria
Berg02_53	*Vibrio sp*. BBsea_05; JF792679	99.5%	*Vibrio*	Gammaproteobacteria
Berg02_64.2	*Vibrio sp*. Mel 35; AJ582806	100.0%	*Vibrio*	Gammaproteobacteria
Berg02_80	*Vibrio sp*. 5C3; EU517625	99.6%	*Vibrio*	Gammaproteobacteria
Berg02_104a	*Vibrio crassostreae*; LGP 8; AJ582809	100.0%	*Vibrio*	Gammaproteobacteria
Berg02_105a	*Vibrio sp*. Mel 35; AJ582806	99.4%	*Vibrio*	Gammaproteobacteria

Table 2 correlates the taxonomic position of the isolates genera and their relative bioactivity percentage. It also specifies the number and relative percentage of bioactive isolates obtained with the *Janus* and Duetz systems. Thirty two isolates of *Alphaproteobacteria* (48.5%) belonging to the genera *Pseudobrivio* and *Labrenzia* were bioactive against *B. subtilis*, *S. aureus* MRSA and *A. fischeri*. Eighteen isolates of *Gammaproteobacteria* (27.3%) belonging to the genera *Vibrio*, *Acinetobacter*, *Microbulbifer* and *Pseudomonas* were active against *B. subtilis*, *V. harveyi* and *A. fischeri*. Eight isolates of *Actinobacteria* (12.1%) belonging to the genera *Gordonia*, *Microbacterium*, *Micrococcus* and *Mycobacterium* were active against *B. subtilis* and *S. aureus* MRSA. Eight isolates of *Firmicutes* (12.1%) belonging to the genera *Bacillus*, *Paenibacillus, Sporosarcina* and *Staphylococcus* were active against *B. subtilis*, *V. harveyi* and *A. fischeri*. *Pseudovibrio* (47.0%), *Vibrio* (22.7%) and *Bacillus* (7.6%) are the three most bioactive genera of all the bioactive isolates. No activity was observed in isolates affiliated to *Ruegeria*, *Rhodobacter*, *Erythrobacter*, *Martelella*, *Nautella*, *Photobacterium*, *Thalassomonas, Rhodococcus* and *Dietzia.*

**Table 2:** Results of bioactivity by genera and screening method (*Janus* and Duetz systems) obtained against *Bacillus subtilis*, *Staphylococcus aureus* MRSA, *Aliivibrio fisheri* and *Vibrio harveyi.*

Phylum/Class	Closest Genus	Number of isolated strains	Number of bioactive strains	% of bioactive strains in the 212 bacteria	Number of bioactive strains in *Janus* system	% of bioactive strains in Janus system	Number of bioactive strains in Duetz system	% of bioactive strains in Duetz system	Number of bioactive strains in both *Janus* and Duetz system
*Actinobacteria*	*Dietzia*	**9**	**0**	**0.00**	**0**	**0.00**	**0**	**0.00**	0
	*Gordonia*	8	3	**4.55**	3	**4.55**	0	**0.00**	0
	*Microbacterium*	3	2	**3.03**	2	**3.03**	0	**0.00**	0
	*Micrococcus*	6	2	**3.03**	2	**3.03**	1	**1.52**	1
	*Mycobacterium*	5	1	**1.52**	1	**1.52**	0	**0.00**	0
	*Rhodococcus*	3	0	**0.00**	0	**0.00**	0	**0.00**	0
	Total of *Actinobacteria*	34	8	**12.12**	8	**12.12**	1	**1.52**	1
*Alphaproteobacteria*	*Pseudovibrio*	61	31	**46.97**	25	**37.88**	9	**13.64**	3
	*Labrenzia*	9	1	**1.52**	1	**1.52**	0	**0.00**	0
	*Ruegeria*	44	0	**0.00**	0	**0.00**	0	**0.00**	0
	*Rhodobacter*	2	0	**0.00**	0	**0.00**	0	**0.00**	0
	*Erythrobacter*	2	0	**0.00**	0	**0.00**	0	**0.00**	0
	*Martelella*	1	0	**0.00**	0	**0.00**	0	**0.00**	0
	*Nautella*	1	0	**0.00**	0	**0.00**	0	**0.00**	0
	Total of *Alphaproteobacteria*	120	32	**48.48**	26	**39.39**	9	**13.64**	2
*Firmicutes*	*Bacillus*	10	5	**7.58**	2	**3.03**	3	**4.55**	0
	*Paenibacillus*	1	1	**1.52**	0	**0.00**	1	**1.52**	0
	*Sporosarcina*	1	1	**1.52**	0	**0.00**	1	**1.52**	0
	*Staphylococcus*	1	1	**1.52**	1	**1.52**	0	**0.00**	0
	Total of *Firmicutes*	13	8	**12.12**	3	**4.55**	5	**7.58**	0
*Gammaproteobacteria*	*Acinetobacter*	2	1	**1.52**	1	**1.52**	0	**0.00**	0
	*Microbulbifer*	7	1	**1.52**	0	**0.00**	1	**1.52**	0
	*Pseudomonas*	6	1	**1.52**	1	**1.52**	0	**0.00**	0
	*Photobacterium*	1	0	**0.00**	0	**0.00**	0	**0.00**	0
	*Thalassomonas*	1	0	**0.00**	0	**0.00**	0	**0.00**	0
	*Vibrio*	28	15	**22.73**	15	**22.73**	0	**0.00**	0
	Total of *Gammaproteobacteria*	45	18	**27.27**	17	**25.76**	1	**1.52**	0
	**Total**	212	66	100.00	54	**81.82**	16	**24.24**	4

The group with the higher number of isolates demonstrating bioactivity was the *Alphaproteobacteria* followed by the *Gammaproteobacteria*, *Actinobacteria* and *Firmicutes*. However, if the analysis is made based on the number of bioactive isolates relative to the total number of bacteria in each group, *Firmicutes* are the most bioactive (61.5%) followed by *Gammaproteobacteria* (40%), *Alphaproteobacteria* (26.7%) and *Actinobacteria* (23.5%). Bioactivity results obtained with marine bacteria and sponge associated bacteria are somehow different. Regarding marine bacteria in general, most of the new marine bacterial compounds from 1997 to 2008 were originated from *Actinobacteria* (40%), *Cyanobacteria* (33%), *Proteobacteria* (12%) and *Firmicutes* and *Bacteroidetes* (5% each) [46]. The distribution of bioactive compounds produced by sponge associated bacteria is *Actinobacteria* (46.66%), *Proteobacteria* (23.33%), *Firmicutes* (11.66%), *Cyanobacteria* (8.33%), *Verrucomicrobia* (5%) and others (5%) [47]. The high number of bioactive *Actinobacteria* may be biased due to their extensive study in the production of antibiotic compounds since 50% of known microbial antibiotics are derived from these bacteria [48].

Several of the obtained bioactive genera are well known producers of metabolites with antimicrobial properties but others are less known. Furthermore, many of the examples referred to below are from sponge associated bacterial isolates.

A suite of antimicrobial compounds with spectra of different antimicrobial activity was observed in *Pseudovibrio* [11]; [49]–[52]. A sponge associated *Alphaproteobacterium* related to *Pseudovibrio denitrificans*, displayed a weak and unstable antimicrobial activity, which was easily lost during cultivation [53]. However, this bioactive bacterium was present in the sponges in high numbers. High antimicrobial activity was also observed in isolates from soft corals affiliated to the alphaproteobacterium *Labrenzia* [51].

Marine vibrios have been reported as a rich source of novel biologically active metabolites [10]; [51]; [54]–[57]. Bioactivity produced by *Vibrio* sp. and sponge extracts was observed against *Bacillus* [58]. A total of 93 secondary metabolites were isolated from *Vibrionaceae* [59]. These are surface-associated bacteria known to produce a broad range of antibacterial compounds which may have a relevant ecological role favouring their abundance in microbial communities [59].

Bioactive metabolites produced by marine *Pseudomonas* species have been reported [10]; [54]; [55]; [60]–[62]. Marine *Pseudomonas* spp. as potential source for medically relevant bioactive substances were revised by Isnansetyo and Kamei [63].

The production of antibacterial compounds by a halotolerant *Acinetobacter* sp. from saltpans of Ribandar, Goa was observed [64]. *Acinetobacter* from the ascidian *Stomozoa murrayi* [65] also produced bioactive compounds. *Microbulbifer* produce anticancer antibiotics (pelagiomicins) [66] and a broad variety of natural parabens [67]. Further evidence of production of antimicrobial activity by *Microbulbifer* was obtained by Penesyan *et al*. [68].

*Bacillus* spp. from terrestrial origin are well known sources of antimicrobial compounds [69]. Similarly antibiotics/bioactive compounds from marine *Bacillus* spp. have also been reported [10]; [53]; [55]–[57]; [70]; [71]. Genome sequencing studies of the genus *Bacillus* have revealed its potential as a source of antibiotic-like compounds [72].

Antimicrobial activity of the Gram negative strains *Paenibacillus* sp., *P. elgii, P. polymyxa* and *P. koreensis* has been observed [70]; [73]–[75]. Anand *et al*. [55] observed bioactivity in *Staphylococcus* bacteria isolated from four species of sponges.

*Micrococcus* strains possessing antimicrobial activity were described by Bultel-Poncé *et al*. [61], Hentschel *et al*. [76] and Lo Giudice *et al*. [77]. Antitumor and antimicrobial bioactivity was observed by *Microbacterium* species [57]; [78]; [79]. In a recent review the actinomycete genus *Gordonia* was described as being capable of degrading xenobiotics, environmental pollutants, or otherwise slowly biodegradable natural polymers as well as to transform or synthesize possibly useful compounds [80]. However, to our knowledge, no antimicrobial activity has been associated with this genus, nor with the *Mycobacterium* genus which is well known for its infectivity.

The most efficient liquid medium for the production of bioactive compounds *against B. subtilis*, *S. aureus* MRSA, *V. harveyi* and *A. fischeri* was MB. In saline R2A bioactivity was observed against both *S. aureus* MRSA and *V. harveyi*, and in saline Starch and MF against *A. fischeri*. In solid media assays, higher numbers of inhibitions were observed in saline Starch followed by MF and saline R2A. No inhibitions were found in MA medium when used as culture medium in the *Janus* system. The non-saline R2A medium was the only one where no bioactive compounds were produced in both systems.

The screening of the 212 bacteria was performed using two different plate systems. The Duetz system is a miniaturized fermentation system that allows carrying out a high number of microfermentations with a lot less effort than the effort required to carry out the fermentations in tubes/flasks. Bacteria are fermented in liquid media, crude extracts are obtained and assayed for bioactivity against target organisms. However, in the double-faced diffusion assay *Janus* system, after the initial growth of the sponge isolates, the medium, containing diffused secondary metabolites is put in contact with the target organism to assess inhibitory responses. The *Janus* system allowed the assessment of a higher number of bioactive positive hits (84%) when compared to the Duetz system (16%). However, the Duetz results are more reliable than the ones obtained with the *Janus* plates due to the overlap of inhibition halos in the latter. Moreover, due to bacterial swarming and gliding, results in the *Janus* system were obtained after 3 days of incubation, whereas longer incubation times were employed in the liquid microfermentations. Bacterial swarming and gliding also interfered with the visualization of results in other antimicrobial studies [37]; [53].

It is well known that microbial secondary metabolite production is highly-dependent on the fermentation conditions [81]. Growth media and growth conditions are variables that are known to have effects on the production of bioactive compounds and can be different depending on the strains [82]. As the production of secondary metabolites is mainly observed in late exponential/stationary phases, bacteria should produce more bioactive compounds after 5 days than 3. This hypothesis could be true for some bacteria and may explain some of the different bioactivity percentages obtained with the two methods. Furthermore, being *Janus* plates assay a microbial culturing system, the production of secondary metabolites is possibly continued along the entire assay at least for some bacteria able to grow at 37°C (incubation temperature of the target microorganisms). Thus some bacteria might be able to produce bioactive compounds in a continuous way for longer while in direct contact with target microorganisms [37]. It is important to notice that assays performed with Duetz systems are cultivated in individual wells, oppositely to the *Janus* assays where the cultivation of the 95 bacteria was performed in the surface of agar without barriers. Therefore, there might be competition for nutrients, growth inhibition due to metabolites produced by the neighbours or even the synergic or antagonistic interaction of two or more bacteria in the production of secondary bioactive metabolites. Thus, this system is not as “clean” as the individual fermentations in Duetz. These aspects can also have some repercussion in the number of hits that were obtained with the two approaches

The search for PKS-I and NRPS genes in 59 of the bioactive bacteria revealed that 30.5% (n = 18) of the bacteria amplified one or two of the genes for secondary metabolites. The presence of PKS-I was observed in 12 strains, NRPS in 3 strains and both PKS-I and NRPS in 3 strains (Table 3). The non-amplification of any of these genes in bioactive bacteria (69.5%) suggests that these strains should amplify with other specific primers for the PKS-I and NRPS genes or use other metabolic pathway for the production of secondary metabolites like PKS-II gene [83].

**Table 3:** Presence of PKS-I and NRPS genes in the bioactive *E. discophorus* bacteria.

Isolate	Closest strain; accession no.	Genera	Gene present
Berg02_22.2	*Gordonia sp*. DEOB200; AY927227	*Gordonia*	PKS-I
Berg02_78	*Gordonia terrae*; 3269aBRRJ; FJ200386	*Gordonia*	NRPS/PKS-I
Berg01_7	*Pseudovibrio ascidiaceicola*	*Pseudovibrio*	PKS-I
Berg01_9	*Pseudovibrio ascidiaceicola* (T); F423( = NBRC 100514); AB175663	*Pseudovibrio*	PKS-I
Berg01_16	*sponge bacterium* Isolate3; AY948383	*Pseudovibrio*	PKS-I
Berg01_33	*alpha proteobacterium* CRA 3GB; AY562562	*Pseudovibrio*	PKS-I
Berg02_8.1	*alpha proteobacterium* CRA 3GB; AY562562	*Pseudovibrio*	PKS-I
Berg02_8.3	*sponge bacterium* Isolate1; AY948382	*Pseudovibrio*	N/D
Berg02_9.1	*alpha proteobacterium* CRA 3GB; AY562562	*Pseudovibrio*	N/D
Berg02_36	*alpha proteobacterium* CRA 3GB; AY562562	*Pseudovibrio*	N/D
Berg02_39.1	*sponge bacterium* Isolate3; AY948383	*Pseudovibrio*	NRPS/PKS-I
Berg02_39.3	*sponge bacterium* Isolate1; AY948382	*Pseudovibrio*	NRPS/PKS-I
Berg02_40	*sponge bacterium* Isolate1; AY948382	*Pseudovibrio*	PKS-I
Berg02_61	*Pseudovibrio ascidiaceicola* (T); F423( = NBRC 100514); AB175663	*Pseudovibrio*	PKS-I
Berg02_63	*sponge bacterium* Isolate1; AY948382	*Pseudovibrio*	N/D
Berg02_65	*Pseudovibrio ascidiaceicola* (T); F423( = NBRC 100514); AB175663	*Pseudovibrio*	N/D
Berg02_141	*sponge bacterium* Isolate1; AY948382	*Pseudovibrio*	PKS-I
Berg02_188	*sponge bacterium* Isolate3; AY948383	*Pseudovibrio*	N/D
Berg01_114	*Bacillus sp*. enrichment culture clone SYW22; FJ601652	*Bacillus*	NRPS
Berg02_161a	*Bacillus sp*. RS654(2010); GU968484	*Bacillus*	NRPS
Berg02_117	*Sporosarcina luteola*	*Sporosarcina*	N/D
Berg02_77	*Pseudomonas sp*. CJ11064; AF500211	*Pseudomonas*	NRPS
Berg02_64.2	*Vibrio sp*. Mel 35; AJ582806	*Vibrio*	PKS-I
Berg02_104a	*Vibrio crassostreae*; LGP 8; AJ582809	*Vibrio*	PKS-I
Berg02_105a	*Vibrio sp*. Mel 35; AJ582806	*Vibrio*	PKS-I

Bacteria that did not amplify for any of the genes are not shown.

N/D – not determined.

The majority of the bioactive bacteria that amplified PKS-I and NRPS genes belongs to the genus *Pseudovibrio* (n = 10). These genes were already reported in *Pseudovibrio* strains isolated from a *Irciniidae* sponge [84]; [85]

Although a high number of the bioactive bacteria belongs to the genus *Vibrio*, only 3 bacteria amplified the PKS-I gene and none the NRPS gene. A recent review on the family *Vibrionaceae* suggests that only NRPS or hybrid PKS-NRPS genes were amplified [59]. Furthermore, the genus *Gordonia* (n = 2), *Bacillus* (n = 2) and *Pseudomonas* (n = 1) amplified the genes NRPS and PKS in lower numbers.

PKS and NRPS genes have been extensively studied in *Actinobacteria* of the genera *Streptomyces*, *Mycobacterium*, *Corynebacterium*, *Micromonospora* [86]–[91] and *Gordonia* [92].

A wide range of bacterial groups were tested for the presence of the genes PKS and NRPS and they were found among other genera in *Pseudomonas*, *Vibrio* and *Bacillus* [93].

These results confirm the production of secondary active metabolites by some *Erylus* strains that are, thus, the most promising ones for future work.

The complex bacterial communities in marine sponges play a considerable ecological role in several aspects of the biology of these organisms, namely by the production of secondary metabolites fundamental for sponge protection against other organisms. These communities have thus a great biotechnological importance in the search for new and more effective pharmaceutical drugs needed for the treatment of severe human diseases such as cancer, microbial infections and inflammatory processes. Our results evidenced the bioactive potential of the heterotrophic bacterial community of the sponge *Erylus discophorus* and open the way for further studies.
